# Fifteen-Year Trends in the Prevalence of Diabetes among Hospitalized HIV-Infected Patients in Spain (1997-2012)

**DOI:** 10.1371/journal.pone.0161953

**Published:** 2016-09-02

**Authors:** Alejandro Alvaro-Meca, Rodrigo Jiménez-Garcia, Isabel Jimenez-Trujillo, Valentin Hernandez-Barrera, Javier de Miguel-Diez, Salvador Resino, Ana Lopez-de-Andres

**Affiliations:** 1 Preventive Medicine and Public Health Teaching and Research Unit, Health Sciences Faculty, Rey Juan Carlos University, Alcorcón, Comunidad de Madrid, Spain; 2 Pneumology Department, Hospital General Universitario Gregorio Marañón, Universidad Complutense de Madrid, Comunidad de Madrid, Spain; 3 Unidad de Infección Viral e Inmunidad. Centro Nacional de Microbiología. Instituto de Salud Carlos III, Madrid, Comunidad de Madrid, Spain; Imperial College London, UNITED KINGDOM

## Abstract

**Objective:**

To describe trends in the prevalence of diabetes among hospitalized HIV-infected patients between 1997 and 2012 in Spain and compare them with those of age- and sex-matched non–HIV-infected patients.

**Methods:**

The study was based on Spanish national hospital discharge data. We performed a retrospective study for the period 1997–2012. HIV infection (HIV-infected versus non–HIV-infected [control group])and calendar period in relation to widespread use of combination antiretroviral therapy (cART) (1997–1999; 2000–2003; 2004–2007 and 2008–2012), were the exposure variables The outcome variables were diagnosis of diabetes and in-hospital mortality (IHM).

**Results:**

From 1997 to 2012, we identified 91,752 cases of diabetes: 15,398 in the HIV-infected group (403,277 hospital admissions) and 76,354 in the non–HIV-infected group (1,503,467 hospital admissions). Overall, HIV-infected patients had lower prevalence values for diabetes than non–HIV-infected patients throughout the follow-up (3.8% vs. 5.1%; p<0.001). The prevalence of diabetes increased 1.56-fold among non–HIV-infected patients and 4.2-fold among HIV-infected patients. The prevalence of diabetes in females was almost twice as high in HIV-infected patients as in non–HIV-infected patients during the last study period (4.72% vs. 2.88%; p<0.001). Diabetes showed a protective effect against IHM throughout the study period (aOR = 0.70; 95%CI, 0.65–0.75).

**Conclusions:**

During the cART era, the prevalence of diabetes has increased sharply among HIV-infected hospitalized patients compared with matched non–HIV-infected subjects. The prevalence of diabetes is rising very fast among HIV-infected women. Diabetes has a protective effect on IHM among HIV-infected patients. Nevertheless, our study has several limitations. No information is available in the database used on important sociodemographic characteristics and relevant clinical variables including duration of the HIV infection, treatments used, drug resistance, treatment adherence or CD4 count, among others. Also, it is possible that increase of diabetes prevalence could reflect the improvement in recording habits.

## Introduction

HIV treatment has improved substantially since the introduction of combination antiretroviral therapy (cART). However, the subsequent improvement in life expectancy has been characterized by an aging HIV-infected population who are increasingly affected by age-related non-communicable diseases [1,2]. Associated comorbidities frequently include metabolic complications that increase the risk of diabetes mellitus. The prevalence of diabetes among HIV-infected patients has been reported to be between 2% and 14% [3–5] and is expected to continue to increase in aging HIV-infected patients.

HIV-infected patients may be at increased risk of developing diabetes as a result of viral coinfection and adverse effects of treatment [6,7]. Previous studies have reported a wide spectrum of metabolic alterations associated with cART, including changes in glucose homeostasis and fat redistribution [8,9]. Protease inhibitors and nucleotide reverse transcriptase inhibitors (NRTIs) have been associated with diabetes [5,10]. Given that the insulin resistance and impaired glucose tolerance induced by cART might act as a precursor of diabetes, the risk of diabetes could have increased in the cART era.

Several studies have reported higher prevalence and/or incidence rates for diabetes in the HIV-infected population than in the general population [11–14], whereas others report similar [6] or even lower [15] rates. After a follow-up of 5.2 years, the DAD study revealed a crude incidence of new diabetes of 4.2 cases per 1000 person-years, which is similar to that described in the Swiss cohort (4.6 cases per 1000 person-years) [16,17]. In Spain, Araujo *et al* [18] reported that the rate of incident diabetes was 2.85 cases per 100 person-years in HIV-infected patients.

To our knowledge, no authors have investigated national trends in the prevalence of diabetes in hospitalized HIV-infected patients or the effect of diabetes on mortality in HIV-infected patients. In the present study, we used national hospital discharge data to describe trends in the prevalence of diabetes among hospitalized HIV-infected patients between 1997 and 2012 in Spain. We compared HIV-infected patients with age- and sex-matched non–HIV-infected patients. We analyzed in-hospital outcomes such as in-hospital mortality (IHM) in patients with and without diabetes and studied the effect of diabetes on mortality among these patients.

## Methods

We performed a retrospective, observational study using the Spanish National Hospital Database (CMBD, *Conjunto Minimo Básico de Datos*, Ministry of Health, Social Services and Equality) which contains data on more than 95% of discharges from public and private hospitals [19], of all consecutive HIV-infected patients aged over 15 years from January 1, 1997 to December 31, 2012.

We subdivided the study period into four calendar periods according to the growing use of cART by HIV-infected patients in Spain [20], as follows: 1997–1999; 2000–2003; 2004–2007; 2008–2012.

A control group of non–HIV-infected patients (selective cohort) admitted to Spanish hospitals was selected in a ratio 4:1 with respect to HIV-infected patients. This approach is useful when the numbers of events is limited and increase the accuracy and power of the statistical test [21–23]. The control group was randomly selected from among HIV-negative patients aged ≥15 years. Patients were matched for gender and age, according to frequency [24], quartiles of age of HIV-infected patients, and assuming an approximate percentage of men, to avoid confounding factors. If a HIV patient was admitted more than once in the study period’s we would only use the data for the first hospital admission. To do this, even if data is anonymous, we could identify the same subject using an algorism including date of birth, sex and province of residence.

We classified diseases and procedures according to the International Classification of Diseases, Ninth Revision, Clinical Modification (ICD-9-CM) [25]. We selected all patients hospitalized with a diagnosis of HIV infection (codes 042 or V08). We classified discharges according to HIV status as randomly selected non-HIV-infected patients and HIV-infected patients.

The primary outcome was presence of diabetes, which was defined as a hospital discharge with a diagnosis of diabetes (any field) (ICD-9-CM codes: 250.XX). The secondary outcomes were length of hospital stay (LOHS) and IHM.

Comorbidity was assessed using the Charlson Comorbidity Index (CCI) excluding diabetes and HIV infection [26]. The prevalence of the diseases included in the CCI in the study groups was analyzed. Finally, information on tobacco use (ICD-9-CM codes 305.1 and V158.2), alcohol abuse (ICD-9-CM codes 305.0, 303.0, 303.9, 291.0, 291.1, 291.2, 291.3, 291.4, 291.5, 291.8, 291.9, 571.0, 571.1, 571.2, 571.3, 425.5, 535.3, 357.5, 265.2, V11.3, 790.3 and 980.0) and other disorders such as obesity (ICD-9-CM 278.00, 278.01, 278.02, 278.03, 278.0, 278.1 and 278.8) and high blood pressure (HBP) (ICD-9-CM 401.XX) were analyzed. According to the CMBD methodology the physician who is discharging the patient fulfills the discharge report only including those diagnosis that, in his opinion, have affected the hospital admission, the duration and outcome.

Continuous variables are presented as means and 95% confidence intervals and for categorical variables as frequencies and percentages. We matched HIV-infected patients and non–HIV-infected patients by frequency, thus enabling statistical tests to be used for independent groups.

We used chi-square or Fisher exact test to analyze categorical data and proportions. We used *t* test or Mann-Whitney test to compare continuous variables. Prevalence was compared using a Poisson distribution. Temporal trends in the prevalence of diabetes were evaluated using a Poisson distribution. We also calculated the odds for prevalence and IHM in patients diagnosed with diabetes according to HIV status using logistic regression models, which were adjusted for age, sex, CCI, obesity and HBP.

Finally, to assess the effect of diabetes on IHM among HIV-infected patients, we used a logistic regression model adjusted for these same variables for each calendar period and for the entire study period. Statistical analyses were performed using package R (version 3.1.2) [27]. Statistical significance was set at p<0.05 (2-tailed).

Data confidentiality was maintained at all times in accordance with Spanish legislation. Patient identifiers were deleted by the Spanish Ministry of Health before the database was provided to the authors in order to maintain patient anonymity. It is not possible to identify patients individually, either in this article or in the database. Since the dataset was anonymous and mandatory, informed consent was unnecessary. The Spanish Ministry of Health considered that our study protocol fulfilled all ethical requirements according to Spanish legislation and provided us with the database. Given the nature of the investigation and according to the Spanish Legislation it is NOT needed the approval of an Ethics Committee S1 Document. Anonymized data was used and authors were NOT involved with the patients' medical treatment nor had any interaction with the participants. None of the authors were affiliated with the hospitals/clinics where patients were treated.

## Results

During the study period, there were 403,277 hospital admissions of HIV-infected patients and 1,503,467 hospital admissions of non–HIV-infected patients. We identified a total of 15,398 hospital admissions of patients with a diagnosis of diabetes in the HIV-infected group from 1997 to 2012 and 76,354 hospital admissions with a diagnosis of diabetes in the non–HIV-infected group.

Table 1 shows the clinical characteristics, risk factors, and outcomes for all hospital admissions due to diabetes according to HIV status during the study period.

**Table 1:** Clinical characteristics, risk factors and outcomes of patients admitted with diabetes stratified by HIV status in Spain, 1997–2012.

	HIV-uninfected patients N = 1,503,467	HIV-infected patients N = 403,277	p-value*
No. of patients with diabetes, N(%)	76,354 (5.07)	15,398 (3.81)	<0.001
Males, N (%)	65,321 (85.6)	12,467 (81)	<0.001
Age (years), Mean (95%CI)	49.05 (48.97–49.14)	50.45 (50.26–50.64)	<0.001
Charlson Comorbidity Index, Mean (95%CI)	1.09 (1.07–1.1)	1.59 (1.57–1.62)	<0.001
Obesity N (%)	9,771 (12.8)	668 (4.3)	<0.001
High Blood Pressure, N (%)	25,262 (33.1)	3,294 (21.4)	<0.001
Alcohol, N (%)	4,233 (5.5)	878 (5.7)	0.447
Tobacco, N (%)	17,173 (22.5)	3,452 (22.4)	0.852
Length of stay (days), Mean (95%CI)	9.1 (9.01–9.19)	11.18 (10.94–11.42)	<0.001
In-hospital mortality, N (%)	2,019 (2.6)	946 (6.1)	<0.001

* Chi-square or Fisher exact test were used to compare categorical data and proportions and t test or Mann-Whitney test to compare continuous variables between HIV-uninfected and infected patients.

Mean age was slightly but significantly higher among HIV-infected than non–HIV-infected patients with diabetes (50.5 years vs. 49.1 years). Males accounted for 85.6% of the non–HIV-infected patients and 81% of the HIV-infected patients (p<0.01).

In comparison with the non–HIV-infected group, the HIV-infected patients had higher IHM, longer LOHS and higher CCI values, whereas non-infected patients had a higher prevalence of HBP and obesity.

Table 2 shows the clinical and epidemiological trends of diabetes in Spain by time period and according to HIV status. Overall, the prevalence of diabetes was lower in the HIV-infected group than in the non–HIV-infected group (3.8% vs. 5.1%; p<0.001). However, the prevalence of diabetes increased sharply among HIV-infected patients, from 1.5% in 1997–1999 to 6.3% in 2008–2012 (p<0.001), and was slightly higher in the HIV-infected group than in the non–HIV-infected group during 2008–2012 (6.3% vs. 6.0%; p<0.001). The prevalence of diabetes rose 1.56 times among non–HIV-infected patients and 4.2 times among HIV-infected patients.

**Table 2:** Clinical and epidemiological trends of hospitalization with diabetes according to HIV status and calendar period in Spain, 1997–2012.

		Calendar Periods				Time trend
		1997–1999	2000–2003	2004–2007	2008–2012	p-value
Total number of admissions	HIV-uninfected	254,919	368,063	389,572	490,913	
	HIV-infected	75,513	104,559	105,016	118,189	
Diabetes diagnosis N (Prevalence)	HIV-uninfected	9789 (3.84)	16600 (4.51)	20475 (5.26)	29490 (6.01)	<0.001
	HIV-infected	1134 (1.5)*	2671 (2.55)*	4121 (3.92)*	7472 (6.32)*	<0.001
Age Mean (95%CI)	HIV-uninfected	48.2 (47.9–48.4)	48.7 (48.5–48.9)	49 (48.9–49.2)	49.6 (49.4–49.7)	<0.001
	HIV-infected	45.1 (44.4–45.8)*	47.5 (47.1–48)*	49.4 (49.1–49.8)	52.9 (52.6–53.1)*	<0.001
Diabetic male N (Prevalence)	HIV-uninfected	8282 (4.51)	14109 (5.36)	17625 (6.43)	25305 (7.32)	<0.001
	HIV-infected	933 (1.66)*	2238 (2.92)*	3396 (4.45)*	5900 (6.95)*	<0.001
Diabetic female N (Prevalence)	HIV-uninfected	1507 (2.12)	2491 (2.37)	2850 (2.47)	4185 (2.88)	<0.001
	HIV-infected	201 (1.04)*	433 (1.56)*	725 (2.53)	1572 (4.72)*	<0.001
Obesity N (Prevalence)	HIV-uninfected	668 (16.36)	1650 (18.78)	2637 (22.75)	4816 (23.01)	<0.001
	HIV-infected	21 (17.8)	62 (18.67)	161 (22.93)	424 (26.43)*	<0.001
High Blood Pressure N (Prevalence)	HIV-uninfected	2151 (15.9)	4727 (18.87)	7035 (21.93)	11349 (23.98)	<0.001
	HIV-infected	84 (15.11)	386 (20.72)	832 (21.85)	1992 (25.03)*	<0.001
Alcohol N (Prevalence)	HIV-uninfected	519 (8.03)	917 (10.75)	1197 (13.28)	1600 (14.98)	<0.001
	HIV-infected	38 (2.65)*	122 (3.96)*	251 (5.79)*	467 (7.94)*	<0.001
Tobacco N (Prevalence)	HIV-uninfected	1428 (6.2)	3255 (7.12)	5010 (8.96)	7480 (9.8)	<0.001
	HIV-infected	115 (1.3)*	476 (2.34)*	915 (3.06)*	1946 (4.94)*	<0.001
In-hospital mortality N (%)	HIV-uninfected	269 (2.75)	449 (2.7)	563 (2.75)	738 (2.5)	0.122
	HIV-infected	77 (6.79)*	179 (6.7)*	253 (6.14)*	437 (5.85)*	0.072
Length of stay Mean (95%CI)	HIV-uninfected	10.2 (10; 10.5)	9.6 (9.4; 9.8)	9.1 (9; 9.3)	8.4 (8.3; 8.6)	<0.001
	HIV-infected	13 (12.2–13.9)*	11.8 (11.3–12.3)*	12.2 (11.6–12.8)*	10.1 (9.8–10.4)*	<0.001
Charlson Comorbidity Index Mean (95%CI)	HIV-uninfected	0.9 (0.9–0.9)	1 (1–1.1)	1.1 (1.1–1.1)	1.2 (1.2–1.2)	<0.001
	HIV-infected	0.9 (0.8–0.9)	1.3 (1.2–1.3)*	1.6 (1.6–1.7)*	1.9 (1.9–1.9)*	<0.001

* Chi-square or Fisher exact test were used to compare categorical data and proportions and t test or Mann-Whitney test to compare continuous variables between HIV-uninfected and infected patients within each time period and in the total period.

Time trend p-values for the prevalence of diabetes were estimated using a Poisson regression model.

Mean age rose significantly from 48.2 to 49.6 years in HIV-infected patients and from 45.1 to 52.9 years among non–HIV-infected patients.

In the HIV-infected group, the prevalence of diabetes increased in both sexes from 1.7% and 1.0% (1997–1999) to 6.9% and 4.7% (2008–2012) in males and females, respectively (p<0.001). The prevalence of diabetes in HIV-infected males was lower than in non–HIV-infected males throughout the study period (p<0.001). However, the prevalence of diabetes in females was almost two times higher in HIV-infected than in non–HIV-infected patients during the last study period (4.7% vs. 2.9%; p<0.001) (Table 2). The prevalence of diabetes was higher among men in all the periods studied, regardless of HIV status.

The prevalence of diabetes was higher in patients with obesity or HBP. In both groups, and for both conditions, the prevalence of diabetes increased over time. Furthermore, in the last period analyzed, the prevalence of diabetes was significantly higher in HIV-infected patients with obesity or HBP than in non–HIV-infected patients (26.4% vs. 23.0% for obesity and 25.0% vs 24% for HBP).

We found the prevalence of diabetes to be higher among non–HIV-infected patients who smoked or drank in all the years analyzed (Table 2).

In-hospital mortality was higher in the HIV-infected group for all four calendar periods: 6.8% vs. 2.8% in 1997–1999 (p<0.001), 6.7% vs. 2.7% in 2000–2003 (p<0.001), 6.1% vs. 2.8% in 2004–2007 (p<0.001) and 5.9% vs. 2.5% in 2008–2012 (p<0.001) (Table 2).

LOHS was also higher in the HIV-infected group in 1997–1999 and 2008–2012; LOHS decreased in both groups from 1997 to 2012 (p<0.001) (Table 2).

**Fig 1:**
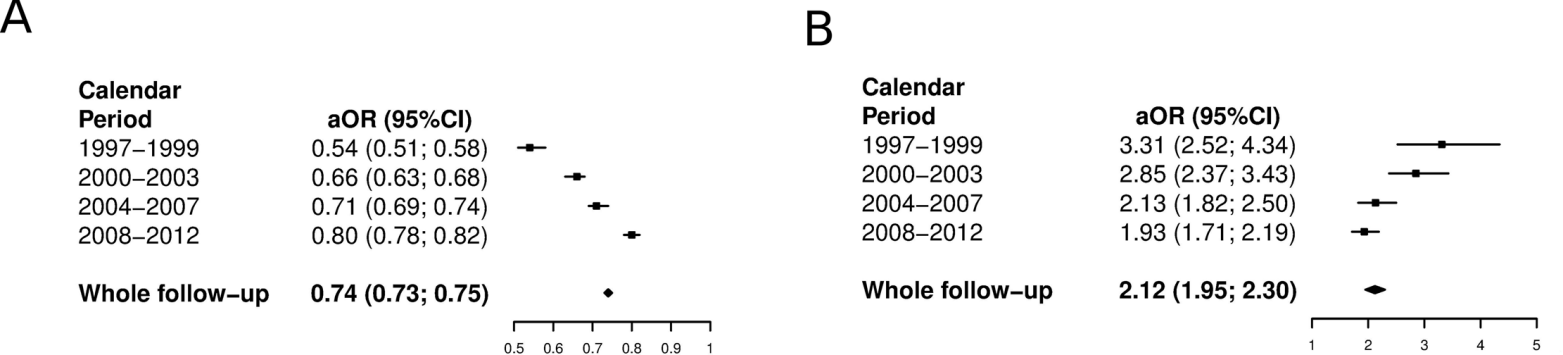
**A. Adjusted likelihood of prevalence among HIV-infected subjects diagnosed with diabetes in Spain (1997–2012) stratified by HIV status. Fig 1B. Adjusted likelihood of death among HIV-infected subjects diagnosed with diabetes in Spain (1997–2012) stratified by HIV status.** (A) Reference category HIV uninfected subjects. Model adjusted by age, sex, CCI, obesity and HBP. (B) Reference category HIV uninfected subjects. Model adjusted by age, sex, CCI, obesity and HBP.

Throughout the study period, the adjusted prevalence for diabetes was 0.74 times lower in the HIV-infected group (Fig 1A). By time period, the adjusted OR (aOR) for prevalence among the HIV-infected group increased when compared with the non–HIV-infected group (aOR = 0.54 in 1997–1999, aOR = 0.66 in 2000–2003, aOR = 0.71 in 2004–2007 and aOR = 0.80 in 2008–2012), suggesting that the adjusted prevalence of diabetes among HIV-infected patients is becoming similar to that found in non–HIV-infected patients (Fig 1A).

The adjusted likelihood of IHM among patients with diabetes was 2.12 times higher in the HIV-infected group throughout the study period (Fig 1B). By time period, the likelihood of death was greater among HIV-infected patients in 1997–1999 (aOR = 3.31), 2000–2003 (aOR = 2.85), 2004–2007 (aOR = 2.13) and in 2008–2012 (aOR = 1.93) (Fig 1B). As with the prevalence of diabetes, the risk of death during hospitalization seems to be increasingly similar over time for both HIV-infected and non–HIV-infected patients.

Table 3 shows the factors associated with IHM among hospitalized HIV-infected patients in Spain from 1997 to 2012 for the total period and each of the calendar periods. Diabetes showed a protective effect for in-hospital mortality throughout the study period (aOR = 0.70; 95%CI, 0.65–0.75) and in each calendar period analyzed.

**Table 3:** Multivariable analysis of the factors associated with mortality among HIV-infected patients in Spain, 1997–2012.

	1997–1999 N = 330,432		2000–2003 N = 472,622		2004–2007 N = 494,588		2008–2012 N = 609,102		Whole follow-up	
	aOR (95%CI)	P-value	aOR (95%CI)	P-value	aOR (95%CI)	P-value	aOR (95%CI)	P-value	aOR (95%CI)	P-value
Diabetes	0.69 (0.54–0.87)	<0.001	0.66 (0.56–0.78)	<0.001	0.65 (0.55–0.75)	<0.001	0.76 (0.68–0.85)	<0.001	0.70 (0.65–0.75)	<0.001
Age	1.02 (1.02–1.03)	<0.001	1.02 (1.02–1.03)	<0.001	1.02 (1.02–1.03)	<0.001	1.02 (1.02–1.03)	<0.001	1.02 (1.02–1.03)	<0.001
Charlson Comorbidity Index	1.40 (1.36–1.44)	<0.001	1.49 (1.46–1.52)	<0.001	1.41 (1.39–1.44)	<0.001	1.32 (1.30–1.35)	<0.001	1.40 (1.39–1.41)	<0.001
Sex (male)	1.44 (1.39–1.55)	<0.001	1.24 (1.16–1.33)	<0.001	1.39 (1.30–1.48)	<0.001	1.24 (1.17–1.32)	<0.001	1.31 (1.27–1.36)	<0.001
Length of stay	1.01 (1.01–1.01)	<0.001	1.01 (1.01–1.01)	<0.001	1.01 (1.01–1.02)	<0.001	1.01 (1.01–1.02)	<0.001	1.01 (1.01–1.01)	<0.001
Calendar Period	N/A		N/A		N/A		N/A			
1997–1999									1	
2000–2003									0.77 (0.74–0.81)	<0.001
2004–2007									0.64 (0.61–0.66)	<0.001
2008–2012									0.51 (0.49–0.54)	<0.001

Older age, higher CCI, male sex, and longer hospital stay were significantly associated with a higher risk of IHM.

Finally, when the calendar period was entered into the model, we observed that the probability of dying during hospitalization decreased significantly by half (aOR = 0.51; 95%CI, 0.49–0.54) from 1997–1999 (reference) to 2008–2012.

## Discussion

Based on the Spanish National Hospital Database, our results reveal that almost 4% of hospitalized HIV-infected patients have an associated diagnosis of diabetes and that the prevalence of diabetes increased significantly from 1997 to 2012. In Italy, Galli *et al* [28] reported that prevalence of diabetes in HIV-infected patients to be 4%, compared with 2.5% in non–HIV-infected patients.

We found that the prevalence of diabetes was lower for HIV-infected patients than for non–HIV-infected patients. In the Veterans Cohort Study in the US, Butt *et al* [29] concluded that the risk of diabetes at baseline was lower in the HIV-infected group than in the non–HIV-infected group (OR, 0.84; 95%CI, 0.72–0.97). The authors also indicated that the net risk of diabetes is determined by a complex interplay of individual factors, with the traditional risk factors (increasing age, minority race and obesity) dominating the profile and leading to an overall lower risk in HIV-infected persons. However our results reveal that, in the following years, the prevalence of diabetes increased sharply among HIV-infected patients, resulting in a higher prevalence of diabetes among HIV-infected patients in the fourth period (2008–2012). One possible explanation is that the use of cART was associated with a significantly higher risk of insulin resistance and diabetes in HIV-infected patients [6,7, 29]. In the Multicenter AIDS Cohort Study (MACS) performed in the US, the prevalence of diabetes adjusted for age and BMI among seropositive patients taking antiretroviral therapy was 4.6 times greater than among seronegative patients (14% in HIV-infected patients vs. 5% in non–HIV-infected patients) [13]. However, in their cohort of 1046 HIV-infected patients, Capeu *et al* [5] found a decreasing trend for the incidence of diabetes mellitus over time, from 23.2 cases/1000 person-years in 1999–2000 to 2.7 cases/1000 person-years in 2005–2006, and concluded that the decline in incidence over time may reflect changes in cART practices and the development of less toxic cART alternatives.

Our investigation reinforces the well-known association between obesity and HBP and diabetes in HIV-infected patients [17,28]. We found that the prevalence of diabetes was higher in patients with obesity or HBP and HIV infection. In a population-based cohort study in Denmark, Rasmussen *et al*. [30] found an increased risk of diabetes in HIV-infected individuals with increasing age (IRR, 4.88 [95%CI, 1.17–20.37] in patients aged 50–59 years; IRR, 10.21 [95%CI, 2.43–42.95] in patients aged >60 years) and BMI (IRR, 9.25 [95%CI 5.37–15.94] in obese patients) and indicated that traditional risk factors seem to be similar in both HIV-infected individuals and the background population. The authors suggested that HIV-infected patients be screened for diabetes in accordance with local general guidelines.

In our study, the prevalence of diabetes in women was almost two times higher in HIV-infected patients than in non–HIV-infected patients in the last time period (2008–2012). In the Women’s Interagency HIV Study, Tien *et al*. [12] reported that among HIV-infected women, longer cumulative exposure to NRTIs was associated with a greater risk of diabetes than no exposure to NRTIs (relative hazard, 2.64 [95%CI, 1.11–6.32] for >3 years’ exposure). In the US, Brar *et al*. [31] found female sex to be significantly associated with diabetes in HIV-infected patients (OR, 2.30; 95%CI, 1.47–3.60) but not in the non–HIV-infected control group. These results suggest that traditional risk factors for diabetes (including loss of ovarian function earlier in life in HIV-infected women than in non–HIV-infected women) appeared to be the most important predictors [32].

The adjusted prevalence of diabetes and the risk of dying from diabetes seem to be similar over time for HIV-infected and non–HIV-infected patients. Our results point to the need for good care of HIV-infected patients based on the assessment of factors such as HIV-related and non–HIV-related comorbidities, patients’ readiness to start cART, and prevention and management of non-infectious comorbidities such as HBP and diabetes [33].

Several factors may explain the protective effect of diabetes on IHM among HIV-infected patients. One explanation is that the presence of diabetes might be an additional factor to be taken into account when selecting the best clinical strategy for these patients. It has been shown that management of HIV-infected patients in accordance with continually updated national and international guidelines and recommendations decreased the risk of death (less than 5% of HIV-infected patients identified during 2007–2012 in Sweden) [33].

Another factor could be the “obesity paradox”—elevated BMI in patients with diabetes could be associated with decreased HIV-related mortality—which has recently been reported in coronary heart disease and other diseases, including incident diabetes [34].

It has been found that HIV infection without satisfactory treatment (as it was in 1997 or before) is associated with lower insulin resistance, that the impact of first HAART was associated with an increased likelihood of survival but also with developing lipodystrophy, and that lipodystrophy was associated with a higher risk of diabetes mellitus [35]. Therefore, diabetes could be a proxy for the bad effects of antiretroviral therapy (lipodystrophy) but also for the good ones (better HIV control and longer survival).

Finally, a possible explanation for this result would be that diabetes may be under-recorded in patients deceased due to severe HIV-related conditions. However we don’t think this should happen more frequently among patients deceased due to severe HIV-related conditions than among those non-HIV patients who died. In our opinion this misclassification bias, is exists, would be non-differential and affect equally both groups therefore, reducing the significance of the association. The main strengths of our study are its large sample size, 15-year follow-up period, and standardized methodology. Nevertheless, our study is subject to a series of limitations.

No information is available in the Spanish Hospital Discharge Database on important sociodemographic characteristics such as race and educational or economical level. Furthermore, relevant clinical variables including duration of the HIV infection, treatments used, drug resistance, treatment adherence or CD4 count, among others, are not included in the database. However we consider that given that we conducted an epidemiological, not clinical investigation, our conclusions are valid and relevant and that most of the residual confounding bias that could be introduced by this clinical variables is controlled by including the CCI as a proxy for the overall health condition of the patients. Also, it is possible that increase of diabetes prevalence could reflect the improvement in recording habits. Ribera et al indicated that as the quality of the coding is apparently gradually improving in Spain [36]. However this improvement would affect equally subjects with and without HIV infection therefore not affecting the conclusions of the investigation. Furthermore, the increase in the age of the study population and the results of other studies conducted in Spain on diabetes prevalence makes us think that the increase in diabetes in our investigation, as a consequence of changes in recording practices, is possibly of a very small magnitude [37–39].

However, beside the limitations of administrative databases for clinical investigation on HIV many studies have used this data sources for relevant epidemiological studies on different aspects of HIV [40–44].

Nevertheless, the CMBD, are periodically audited and the validity of the “*diabetes diagnosis*” in hospital discharge reports has been demonstrated in the past [36, 45–47].

In conclusion, we found that in the cART era, the prevalence of diabetes has increased sharply among HIV-infected patients compared with age- and sex-matched non–HIV-infected patients. The prevalence of diabetes is rising very fast among HIV-infected women. Diabetes has a protective effect against IHM among HIV-infected patients.

Our findings suggest that diabetes is a health problem that affects HIV-infected and non–HIV-infected patients in much the same way.

## Supporting Information

S1 Document No need for approval of an Ethics Committee document. (PDF)

## Abbreviations

cART: combination antiretroviral therapy
CCI: Charlson Comorbidity Index
HBP: high blood pressure
ICD-9-CM: International Classification of Diseases, Ninth Revision, Clinical Modification
IHM: in-hospital mortality
LOHS: length of hospital stay
MBDS: Minimum Basic Data Set
NRTIs: nucleotide reverse transcriptase inhibitors
