# Supplementary material for: Fifteen-Year Trends in the Prevalence of Diabetes among Hospitalized HIV-Infected Patients in Spain (1997-2012)
Source: PLoS One. 2016 Sep 2;11(9):e0161953. doi: 10.1371/journal.pone.0161953 (PMC5010187; doi:10.1371/journal.pone.0161953)
Supplement: S1 Document — (PDF) [file pone.0161953.s001.pdf]

Universidad
Rey Juan Carlos

Rectorado

D. José Luis del Barrio Fernández, como Secretario del Comité de Ética de la Investigación de la Universidad Rey Juan Carlos

CERTIFICA que:

El Proyecto de Investigación con el título: **"INCIDENCIA Y CARACTERÍSTICAS DE LAS ENFERMEDADES Y PROCEDIMIENTOS EN LAS HOSPITALIZACIONES DE LOS INDIVIDUOS CON DIABETES MELLITUS EN ESPAÑA. EVOLUCIÓN TEMPORAL, ANÁLISIS POR COMORBILIDAD Y GÉNERO"**, dirigido por la Dra. Doña Ana López de Andrés, **NO NECESITA** certificado del Comité Ética de la Investigación dada la naturaleza de dicha investigación, al no encontrarse dentro de los supuestos establecidos en el artículo 2.e) de la Ley 14/2007, de 3 de junio, de Investigación Biomédica

Móstoles, 28 de Junio de 2013

UNIVERSIDAD REY JUAN CARLOS
MCMXCVI
COMITÉ DE ÉTICA DE LA INVEST

Fdo.: José Luis del Barrio Fernández

Tulipán, s/n E 28933 Móstoles Madrid España Tel. 34 91 665 50 60 Fax 34 91 614 71 20
